# Novel thiazolidinone-containing compounds, without the well-known sulphonamide zinc-binding group acting as human carbonic anhydrase IX inhibitors

**DOI:** 10.1080/14756366.2018.1499628

**Published:** 2018-09-24

**Authors:** Özlen Güzel-Akdemir, Andrea Angeli, Kübra Demir, Claudiu T. Supuran, Atilla Akdemir

**Affiliations:** a Department of Pharmaceutical Chemistry, Faculty of Pharmacy, Istanbul University, Istanbul, Turkey;; b Department of NEUROFARBA, Sezione di Scienze Farmaceutiche Universita degli Studi di Firenze, Sesto Fiorentino, Florence, Italy;; c Computer-Aided Drug Discovery Laboratory, Department of Pharmacology, Faculty of Pharmacy, Bezmialem Vakif University, Istanbul, Turkey

**Keywords:** carbonic anhydrase IX, hCA IX, thiazolidinone, molecular modelling, docking

## Abstract

A small collection of 26 structurally novel thiazolidinone-containing compounds, without the well-known sulphonamide zinc-binding group, were synthesised and tested in enzyme inhibition assays against the tumour-associated hCA IX enzyme. Inhibition constants in the lower micromolar region (*K*_I_ < 25 μM) have been measured for 17 of the 26 compounds. Even though the *K*_I_ values are relatively weak, the fact that they do not contain a sulphonamide moiety suggests that these compounds do not interact with the active site zinc ion. Therefore, docking studies and molecular dynamics simulations have been performed to suggest binding poses for these structurally novel inhibitors.

## Introduction

Carbonic anhydrases (CAs; EC 4.2.1.1) are a structurally diverse family of metallo-enzymes that catalyse the reversible hydration of carbon dioxide to bicarbonate and protons^1^. Due to this physiologically important reaction, CAs are important in controlling pH values and supplying bicarbonate ions for various processes. The human CA isoform IX (hCA IX), which belongs to the α-subfamily of CAs (αCAs), is expressed in the stomach and peritoneal lining. More interestingly, hCA IX is upregulated in many solid hypoxic tumours and it helps the tumour cell to function in an acidic environment^1–5^. This provides an advantage over healthy cells in the tumour microenvironment. As such, the development of selective hCA IX inhibitors may provide novel compounds for the cancer chemotherapy.

Many inhibitors of hCA IX has been synthesised and tested in the last decade, including substituted-phenylacetamido aromatic sulphonamides, open saccharin analogues, probenecid analogues, and isatin analogues^5–11^. Many CA inhibitors (including hCA IX inhibitors) bind to the active site zinc ion via a so called zinc-binding group (ZBG), which is a sulphonamide in most cases, and thereby block the reversible hydration of carbon dioxide^12^. One such example is compound SLC-0111, a sulphonamide-containing potent CA inhibitor, which is currently in clinical trials^13–21^. Other binding modes of inhibitors are also possible, including binding to allosteric sites and binding to the active site but without a direct interaction with the zinc ion^12^.

Recently, we focussed our attention to the 4-thiazolidinone moiety, which in general provides good safety profiles, bioavailabilities, and various biological activities^22^. Several studies report that thiazolidinone-based agents show diverse pharmacological properties including antifungal^23^, antiparasitic^24^, antimicrobial^25^, antioxidant^26^, anticonvulsant^27^, anti-HIV^28^, anti-inflammatory^29^, anti-tuberculosis^30^, and anti-tumour activities^31^.

In this work, we synthesised and tested 26 structurally novel thiazolidinone-containing compounds, without the well-known sulphonamide ZBG, in enzyme inhibition assays against hCA IX. Inhibition constants were obtained in the lower micromolar region. Subsequently, docking studies and molecular dynamics simulations were performed to suggest binding poses for these compounds.

## Materials and methods

### Chemistry

All reagents and solvents were purchased from Sigma-Aldrich (Darmstadt, Germany) and used without further purification. Melting points were measured in open capillary tubes with a Buchi 530 melting point apparatus and are uncorrected. IR (KBr) spectra were recorded using a Perkin-Elmer 1600 FTIR spectrophotometer. ^1^H-NMR, HSQC, and ^13^C-NMR (proton decoupled) spectra were recorded on a Varian^UNITY^INOVA 500 MHz spectrometer and Agilent VNMRS 600 MHz NMR spectrometer. Elemental analyses were performed on a Carlo Erba Model 1106 elemental analyser. Mass spectra (LC/MS-APCI) were recorded on a Finnigan^TM^ LCQ^TM^ Mass Spectrometer in the negative ionisation mode.

#### (RS)-2-hydroxy-2-phenylacetohydrazide (1)^32^^,^^33^

(*RS*)-methyl 2-hydroxy-2-phenylacetate (0.05 mol) and 12 ml hydrazine hydrate (98%) were heated under reflux for 12 h. The reaction mixture was transferred to a crystallising dish and left aside until crystallisation. The crude product thus obtained was recrystallised from ethanol.

#### General procedure for synthesis of (RS)-1–(2-hydroxy-2-phenylacetyl)-4-substituted thiosemicarbazides (2)^32^

To a solution of 0.005 mol **1** in 30 ml EtOH, 0.005 mol of an appropriate isothiocyanate were added. The resulting mixture was heated under reflux for 3 h. After cooling, the precipitate was separated and purified by washing with hot EtOH or recrystallisation from EtOH.

#### (RS)-1–(2-hydroxy-2-phenylacetyl)-4–(2-bromophenyl)thiosemicarbazide (2g)

Yellow powder, m.p. 154.1–154.4 °C, 70% yield; IR (KBr) (υ, cm^−1^), 3307, 3267 (OH/NH), 1699 (C=O); ^1^H-NMR (DMSO-d_6_, 600 MHz) δ (ppm): 5.11 (1H, d, *J* = 4.40 Hz, C*H*OH), 6.06 (1H, brs, CHO*H*), 7.17 (1H, t, *J =* 7.70 Hz, Ar-H), 7.27 (1H, t, *J* = 7.40 Hz, Ar-H), 7.31–7.34 (2H, m, Ar-H), 7.37 (1H, t, *J* = 7.70 Hz, Ar-H), 7.43 (1H, d, *J =* 8.40 Hz, Ar-H), 7.49 (1H, d, *J =* 7.10 Hz, Ar-H), 7.60 (1H, dd, *J =* 8.10, 1.60 Hz, Ar-H), 7.64 (1H, dd, *J =* 8.00, 1.40 Hz, Ar-H), 9.05 (1H, s, N_4_H), 9.79 (1H, s, N_2_H), 10.35 (1H, s, N_1_H). Anal. Calcd. for C_15_H_14_BrN_3_O_2_S (380.259): C, 47.38; H, 3.71; N, 11.05; S, 8.43. Found: C, 47.67; H, 3.55; N, 11.36; S, 8.71.

#### (RS)-1–(2-hydroxy-2-phenylacetyl)-4–(3-bromophenyl)thiosemicarbazide (2h)

Light yellow powder, m.p. 135.5–135.7 °C, 85% yield; IR (KBr) (υ, cm^−1^), 3404, 3282 (OH/NH), 1699 (C=O); ^1^H-NMR (DMSO-d_6_, 500 MHz) δ (ppm): 5.11 (1H, d, *J* = 4.10 Hz, C*H*OH), 6.08 (1H, brs, CHO*H*), 7.28 (2H, q, *J =* 7.70 Hz, Ar-H), 7.34 (3H, t, *J* = 7.40 Hz, Ar-H), 7.48 (3H, d, *J* = 7.50 Hz, Ar-H), 7.73 (1H, s, Ar-H), 9.33 (1H, s, N_4_H), 9.81 (1H, s, N_2_H), 10.26 (1H, s, N_1_H). Anal. Calcd. for C_15_H_14_BrN_3_O_2_S (380.259): C, 47.38; H, 3.71; N, 11.05; S, 8.43. Found: C, 47.07; H, 3.35; N, 11.42; S, 8.55.

#### General procedure for synthesis of (RS)-N'-[3–(4-substitutedphenyl)-4-oxo-1,3-thiazolidin-2-ylidene]-2-hydroxy-2-phenylacetohydrazide (3)/(RS)-N'-[3–(4-substitutedphenyl)-5-methyl-4-oxo-1,3-thiazolidin-2-ylidene]-2-hydroxy-2-phenylacetohydrazide (4)^34^

A mixture of 0.005 mol **2**, 0.005 mol of ethyl α-bromoacetate/ethyl α-bromopropionate, and 0.02 mol of fused sodium acetate in 25 ml anhydrous EtOH was heated under reflux for 3 h. The reaction mixture was cooled, diluted with water, and allowed to stand overnight. The precipitate was filtered, dried, and recrystallised from EtOH.

#### (RS)-N'-[3–(2-fluorophenyl)-4-oxo-1,3-thiazolidin-2-ylidene]-2-hydroxy-2-phenylacetohydrazide (3a)

White flakes, m.p. 182–185 °C, 77% yield; IR (KBr) (υ, cm^−1^), 3316 (OH/NH), 1696, 1754 (C=O); ^1^H-NMR (DMSO-d_6_, 500 MHz) δ (ppm): 4.20 (2H, s, H5-thz), 5.18 (1H, s, C*H*OH), 6.49 (1H, s, CHO*H*), 6.93 (1H, t, *J =* 8.78 Hz, Ar-H), 7.15–7.20 (2H, m, Ar-H), 7.25–7.32 (4H, m, Ar-H), 7.53 (2H, d, *J =* 6.84 Hz, Ar-H), 10.96 (1H, brs, CONH). Anal. Calcd. for C_17_H_14_FN_3_O_3_S (359.376): C, 56.82; H, 3.93; N, 11.69; S, 8.92. Found: C, 56.48; H, 3.55; N, 11.86; S, 9.01.

#### (RS)-N'-[3–(3-fluorophenyl)-4-oxo-1,3-thiazolidin-2-ylidene]-2-hydroxy-2-phenylacetohydrazide (3b)

White crystal, m.p. 209–211 °C, 68% yield; IR (KBr) (υ, cm^−1^), 3293 (OH/NH), 1692, 1766 (C=O); ^1^H-NMR (DMSO-d_6_, 500 MHz) δ (ppm): 4.19 (2H, s, H5-thz), 5.18 (1H, s, C*H*OH), 6.46 (1H, s, CHO*H*), 6.92–6.95 (1H, m, Ar-H), 7.15–7.19 (2H, m, Ar-H), 7.24–7.32 (4H, m, Ar-H), 7.53 (2H, d, *J =* 6.84 Hz, Ar-H), 10.93 (1H, brs, CONH). Anal. Calcd. for C_17_H_14_FN_3_O_3_S (359.376): C, 56.82; H, 3.93; N, 11.69; S, 8.92. Found: C, 57.22; H, 3.90; N, 11.95; S, 9.03.

#### (RS)-N'-[3–(4-fluorophenyl)-4-oxo-1,3-thiazolidin-2-ylidene]-2-hydroxy-2-phenylacetohydrazide (3c)

White crystal, m.p. 211–213 °C, 75% yield; IR (KBr) (υ, cm^−1^), 3286, 3567 (OH/NH), 1689, 1763 (C=O); ^1^H-NMR (DMSO-d_6_, 500 MHz) δ (ppm): 4.16 (2H, s, H5-thz), 5.19 (1H, d, *J =* 1.96 Hz, C*H*OH), 6.48 (1H, d, *J =* 3.90 Hz, CHO*H*), 6.88 (2H, d, *J =* 4.88 Hz, Ar-H), 7.20 (2H, t, *J =* 7.68 Hz, Ar-H), 7.29–7.33 (3H, m, Ar-H), 7.54 (2H, d, *J =* 7.32 Hz, Ar-H), 10.84 (1H, s, CONH). LC/MS: *m/z* 358 (M-H)^-^. Anal. Calcd. for C_17_H_14_FN_3_O_3_S (359.376): C, 56.82; H, 3.93; N, 11.69; S, 8.92. Found: C, 56.94; H, 4.05; N, 11.90; S, 8.58.

#### (RS)-N'-[3–(2-chlorophenyl)-4-oxo-1,3-thiazolidin-2-ylidene]-2-hydroxy-2-phenylacetohydrazide (3d)

Ivory powder, m.p. 183–185 °C, 74% yield; IR (KBr) (υ, cm^−1^), 3320 (OH/NH), 1698, 1753 (C=O); ^1^H-NMR (DMSO-d_6_, 500 MHz) δ (ppm): 4.20 (2H, s, H5-thz), 5.20 (1H, s, C*H*OH), 6.49 (1H, brs, CHO*H*), 6.92 (1H, dd, *J =* 7.81, 1.46 Hz, Ar-H), 7.15 (1H, td, *J =* 7.81, 1.46 Hz, Ar-H), 7.29–7.32 (4H, m, Ar-H), 7.49 (1H, dd, *J =* 7.81, 1.46 Hz, Ar-H), 7.54 (2H, dd, *J =* 6.84, 0.98 Hz, Ar-H), 10.96 (1H, brs, CONH). ^13^C-NMR (proton decoupled, DMSO-d_6_, 150 MHz) δ (ppm): 30.05 (thz. C5), 72.97 (CH-OH), 120.24, 121.68, 122.84, 125.68, 127.47, 127.66, 127.73, 127.93, 127.99 (Ar-CH), 130.00, 140.41, 144.71 (Ar-C), 153.75 (thz. C2), 168.31 (CONH), 170.50 (thz. C4). Anal. Calcd. for C_17_H_14_ClN_3_O_3_S (375.830): C, 54.33; H, 3.75; N, 11.18; S, 8.53. Found: C, 54.02; H, 3.55; N, 11.23; S, 8.44.

#### (RS)-N'-[3–(3-chlorophenyl)-4-oxo-1,3-thiazolidin-2-ylidene]-2-hydroxy-2-phenylacetohydrazide (3e)

Ivory powder, m.p. 202–204 °C, 77% yield; IR (KBr) (υ, cm^−1^), 3276 (OH/NH), 1689, 1765 (C=O); ^1^H-NMR (DMSO-d_6_, 500 MHz) δ (ppm): 4.19 (2H, s, H5-thz), 5.19 (1H, d, *J =* 3.42 Hz, C*H*OH), 6.49 (1H, d, *J =* 4.39 Hz, CHO*H*), 6.84 (2H, d, *J =* 7.81 Hz, Ar-H), 7.20 (1H, dt, *J =* 8.30,1.46 Hz, Ar-H), 7.29–7.33 (3H, m, Ar-H), 7.40 (1H, t, *J =* 8.05 Hz, Ar-H), 7.54 (2H, d, *J =* 7.33 Hz, Ar-H), 10.84 (1H, s, CONH). Anal. Calcd. for C_17_H_14_ClN_3_O_3_S (375.830): C, 54.33; H, 3.75; N, 11.18; S, 8.53. Found: C, 54.67; H, 3.46; N, 11.23; S, 8.48.

#### (RS)-N'-[3–(4-chlorophenyl)-4-oxo-1,3-thiazolidin-2-ylidene]-2-hydroxy-2-phenylacetohydrazide (3f)

Ivory powder, m.p. 221–222 °C, 70% yield; IR (KBr) (υ, cm^−1^), 3280 (OH/NH), 1689, 1765 (C=O); ^1^H-NMR (DMSO-d_6_, 500 MHz) δ (ppm): 4.18 (2H, s, H5-thz), 5.18 (1H, s, C*H*OH), 6.48 (1H, brs, CHO*H*), 6.88 (2H, d, *J =* 8.30 Hz, Ar-H), 7.29–7.33 (3H, m, Ar-H), 7.42 (2H, d, *J =* 6.34 Hz, Ar-H), 7.53 (2H, d, *J =* 7.32 Hz, Ar-H), 10.86 (1H, s, CONH). ^13^C-NMR (HSQC-2D, DMSO-d_6_, 125 MHz) δ (ppm): 30.63 [thz. C5 – 4.18 ppm (2H, s, SCH_2_)], 73.72 [**C**HOH – 5.18 ppm (1H, s, C**H**OH)], 123.24 [Ar-**C**H – 6.88 ppm (2H, d, *J* = 8.30 Hz, Ar-H)], 127.97 [Ar-**C**H – 7.53 ppm (2H, d, *J* = 7.32 Hz, Ar-H)], 128.39 [Ar-**C**H – 7.29–7.33 (3H, m, Ar-H)], 130.03 [Ar-**C**H – 7.42 ppm (2H, d, *J* = 6.34 Hz, Ar-H)]. ^13^C-NMR (proton decoupled, DMSO-d_6_, 150 MHz) δ (ppm): 29.92 (thz. C5), 72.95 (CH-OH), 122.52, 127.17, 127.33, 127.69, 127.83, 127.95, 128.45, 129.32 (Ar-CH), 140.41, 146.20, 146.35 (Ar-C), 152.51 (thz. C2), 168.24 (CONH), 170.68 (thiaz. C4). Anal. Calcd. for C_17_H_14_ClN_3_O_3_S (375.830): C, 54.33; H, 3.75; N, 11.18; S, 8.53. Found: C, 54.48; H, 3.79; N, 11.19; S, 8.18.

#### (RS)-N'-[3–(2-bromophenyl)-4-oxo-1,3-thiazolidin-2-ylidene]-2-hydroxy-2-phenylacetohydrazide (3g)

White powder, m.p. 167–169 °C, 61% yield; IR (KBr) (υ, cm^−1^), 3331 (OH/NH), 1699, 1752 (C=O); ^1^H-NMR (DMSO-d_6_, 500 MHz) δ (ppm): 4.20 (2H, s, H5-thz), 5.19 (1H, d, *J =* 2.92 Hz, C*H*OH), 6.47 (1H, d, *J =* 3.90 Hz, CHO*H*), 6.92 (1H, dd, *J =* 7.81, 1.46 Hz, Ar-H), 7.07 (1H, td, *J =* 7.81, 1.46 Hz, Ar-H), 7.29–7.34 (3H, m, Ar-H), 7.36 (1H, t, *J =* 6.83 Hz, Ar-H), 7.55 (2H, d, *J =* 6.83 Hz, Ar-H), 7.65 (1H, d, *J =* 8.05 Hz, Ar-H), 10.93 (1H, s, CONH). Anal. Calcd. for C_17_H_14_BrN_3_O_3_S (420.281): C, 48.58; H, 3.36; N, 10.00; S, 7.63. Found: C, 48.18; H, 3.28; N, 9.86; S, 8.01.

#### (RS)-N'-[3–(3-bromophenyl)-4-oxo-1,3-thiazolidin-2-ylidene]-2-hydroxy-2-phenylacetohydrazide (3h)

White powder, m.p. 199–201 °C, 74% yield; IR (KBr) (υ, cm^−1^), 3272, 3566 (OH/NH), 1689, 1765 (C=O); ^1^H-NMR (DMSO-d_6_, 500 MHz) δ (ppm): 4.19 (2H, s, H5-thz), 5.19 (1H, d, *J =* 3.91 Hz, C*H*OH), 6.47 (1H, d, *J =* 4.88 Hz, CHO*H*), 6.88 (1H, s, Ar-H), 6.97 (1H, brs, Ar-H), 7.28–7.36 (5H, m, Ar-H), 7.53 (2H, d, *J =* 7.32 Hz, Ar-H), 10.86 (1H, s, CONH). Anal. Calcd. for C_17_H_14_BrN_3_O_3_S (420.281): C, 48.58; H, 3.36; N, 10.00; S, 7.63. Found: C, 48.78; H, 3.37; N, 9.58; S, 7.49.

#### (RS)-N'-[3–(4-bromophenyl)-4-oxo-1,3-thiazolidin-2-ylidene]-2-hydroxy-2-phenylacetohydrazide (3i)

White powder, m.p. 216–218 °C, 70% yield; IR (KBr) (υ, cm^−1^), 3276 (OH/NH), 1688, 1763 (C=O); ^1^H-NMR (DMSO-d_6_, 500 MHz) δ (ppm): 4.18 (2H, s, H5-thz), 5.18 (1H, s, C*H*OH), 6.48 (1H, s, CHO*H*), 6.82 (2H, d, *J =* 7.81 Hz, Ar-H), 7.29–7.33 (3H, m, Ar-H), 7.52–7.56 (4H, m, Ar-H), 10.86 (1H, s, CONH). Anal. Calcd. for C_17_H_14_BrN_3_O_3_S (420.281): C, 48.58; H, 3.36; N, 10.00; S, 7.63. Found: C, 48.64; H, 3.35; N, 9.56; S, 7.63.

#### (RS)-N'-[3–(2-methylphenyl)-4-oxo-1,3-thiazolidin-2-ylidene]-2-hydroxy-2-phenylacetohydrazide (3j)

Light yellow powder, m.p. 188–190 °C, 67% yield; IR (KBr) (υ, cm^−1^), 3343 (OH/NH), 1705, 1749 (C=O); ^1^H-NMR (DMSO-d_6_, 500 MHz) δ (ppm): 1.91 (3H, d, *J =* 13.66 Hz, -CH_3_), 4.14 (2H, s, H5-thz), 5.19 (1H, s, C*H*OH), 6.50 (1H, s, CHO*H*), 6.75 (1H, dd, *J =* 7.81, 0.98 Hz, Ar-H), 7.02 (1H, t, *J =* 7.32 Hz, Ar-H), 7.14–7.19 (2H, m, Ar-H), 7.26–7.36 (3H, m, Ar-H), 7.53 (2H, d, *J =* 7.81 Hz, Ar-H), 10.90 (1H, s, CONH). Anal. Calcd. for C_18_H_17_N_3_O_3_S (355.412): C, 60.83; H, 4.82; N, 11.82; S, 9.02. Found: C, 60.94; H, 4.90; N, 12.05; S, 9.20.

#### (RS)-N'-[3–(3-methylphenyl)-4-oxo-1,3-thiazolidin-2-ylidene]-2-hydroxy-2-phenylacetohydrazide (3k)

Ivory powder, m.p. 205–207 °C, 80% yield; IR (KBr) (υ, cm^−1^), 3284 (OH/NH), 1690, 1762 (C=O); ^1^H-NMR (DMSO-d_6_, 500 MHz) δ (ppm): 2.29 (3H, s, -CH_3_), 4.13 (2H, s, H5-thz), 5.18 (1H, s, C*H*OH), 6.46 (1H, brs, CHO*H*), 6.66 (2H, d, *J =* 7.81 Hz, Ar-H), 6.95 (1H, d, *J =* 7.81 Hz, Ar-H), 7.24 (1H, t, *J =* 7.81 Hz, Ar-H), 7.28–7.33 (3H, m, Ar-H), 7.55 (2H, d, *J =* 7.32 Hz, Ar-H), 10.85 (1H, brs, CONH). Anal. Calcd. for C_18_H_17_N_3_O_3_S (355.412): C, 60.83; H, 4.82; N, 11.82; S, 9.02. Found: C, 60.57; H, 4.85; N, 11.79; S, 8.93.

#### (RS)-N'-[3–(4-methylphenyl)-4-oxo-1,3-thiazolidin-2-ylidene]-2-hydroxy-2-phenylacetohydrazide (3l)

White powder, m.p. 197–199 °C, 75% yield; IR (KBr) (υ, cm^−1^), 3226 (OH/NH), 1692, 1763 (C=O); ^1^H-NMR (DMSO-d_6_, 500 MHz) δ (ppm): 2.28 (3H, s, -CH_3_), 4.13 (2H, s, H5-thz), 5.17 (1H, s, C*H*OH), 6.48 (1H, brs, CHO*H*), 6.75 (2H, d, *J =* 8.29 Hz, Ar-H), 7.16 (2H, d, *J =* 8.30 Hz, Ar-H), 7.29–7.33 (3H, m, Ar-H), 7.54 (2H, d, *J =* 6.83 Hz, Ar-H), 10.85 (1H, s, CONH). Anal. Calcd. for C_18_H_17_N_3_O_3_S (355.412): C, 60.83; H, 4.82; N, 11.82; S, 9.02. Found: C, 60.64; H, 5.00; N, 12.05; S, 9.01.

#### (RS)-N'-[3–(4-methoxyphenyl)-4-oxo-1,3-thiazolidin-2-ylidene]-2-hydroxy-2-phenylacetohydrazide (3m)

Ivory powder, m.p. 186–188 °C, 61% yield; IR (KBr) (υ, cm^−1^), 3269 (OH/NH), 1689, 1763 (C=O); ^1^H-NMR (DMSO-d_6_, 500 MHz) δ (ppm): 3.75 (3H, s, OCH_3_), 4.13 (2H, s, H5-thz), 5.18 (1H, s, C*H*OH), 6.46 (1H, brs, CHO*H*), 6.82 (2H, d, *J =* 6.84 Hz, Ar-H), 6.93 (2H, d, *J =* 6.59 Hz, Ar-H), 7.28–7.34 (3H, m, Ar-H), 7.55 (2H, d, *J =* 7.33 Hz, Ar-H), 10.81 (1H, s, CONH). 13 C-NMR (HSQC-2D, DMSO-d_6_, 125 MHz) δ (ppm): 30.48 [thz. C5 – 4.13 ppm (2H, s, SCH_2_)], 55.92 [O**C**H_3_ – 3.75 (3H, s, OC**H**_3_)], 73.74 [**C**HOH – 5.18 ppm (1H, s, C**H**OH)], 115.25 [Ar-**C**H – 6.93 ppm (2H, d, *J* = 6.59 Hz, Ar-H)], 122.54 [Ar-**C**H – 6.82 ppm (2H, d, *J* = 6.84 Hz, Ar-H)], 128.01 [Ar-**C**H – 7.55 ppm (2H, d, *J* = 7.33 Hz, Ar-H)], 128.64 [Ar-**C**H – 7.28–7.34 (3H, m, Ar-H)]. ^13^C-NMR (proton decoupled, DMSO-d_6_, 150 MHz) δ (ppm): 29.76 (thz. C5), 73.07 (CH-OH), 114.17, 114.52, 118.34, 121.82, 127.21, 127.40, 127.67, 127.94, 134.83 (Ar-CH), 140.49, 150.85, 156.177 (Ar-C), 153.82 (thz. C2), 168.22 (CONH), 170.64 (thz. C4). Anal. Calcd. for C_18_H_17_N_3_O_4_S (371.411): C, 58.21; H, 4.61; N, 11.31; S, 8.63. Found: C, 58.54; H, 4.30; N, 11.47; S, 8.20.

#### (RS)-N'-[3–(4-nitrophenyl)-4-oxo-1,3-thiazolidin-2-ylidene]-2-hydroxy-2-phenylacetohydrazide (3n)

Orange crystal, m.p. 174–176 °C, 67% yield; IR (KBr) (υ, cm^−1^), 3281 (OH/NH), 1689, 1763 (C=O); ^1^H-NMR (DMSO-d_6_, 500 MHz) δ (ppm): 4.24 (2H, s, H5-thz), 5.19 (1H, s, C*H*OH), 6.50 (1H, brs, CHO*H*), 7.07–7.08 (2H, m, Ar-H), 7.30–7.33 (3H, m, Ar-H), 7.53 (2H, d, *J =* 7.81 Hz, Ar-H), 8.25 (2H, d, *J =* 8.05 Hz, Ar-H), 11.03 (1H, brs, CONH). Anal. Calcd. for C_17_H_14_N_4_O_5_S (386.383): C, 52.84; H, 3.65; N, 14.50; S, 8.30. Found: C, 52.67; H, 3.54; N, 14.67; S, 8.59.

#### (RS)-N'-[3–(3-trifluoromethylphenyl)-4-oxo-1,3-thiazolidin-2-ylidene]-2-hydroxy-2-phenylacetohydrazide (3o)

Light yellow crystal, m.p. 208–210 °C, 75% yield; IR (KBr) (υ, cm^−1^), 3305 (OH/NH), 1692, 1756 (C=O); ^1^H-NMR (DMSO-d_6_, 500 MHz) δ (ppm): 4.20 (2H, s, H5-thz), 5.19 (1H, s, C*H*OH), 6.47 (1H, s, CHO*H*), 7.02–7.10 (1H, m, Ar-H), 7.18 (1H, d, *J =* 7.81 Hz, Ar-H), 7.30–7.33 (3H, m, Ar-H), 7.50 (1H, d, *J =* 7.81 Hz, Ar-H), 7.54 (2H, d, *J =* 7.33 Hz, Ar-H), 7.62 (1H, t, *J =* 7.81 Hz, Ar-H), 10.90 (1H, brs, CONH). ^13^C-NMR (proton decoupled, DMSO-d_6_, 150 MHz) δ (ppm): 30.03 (thz. C5), 73.08 (CH-OH), 123.90 (q, *J* = 271.05 Hz, CF_3_), 130.10 (q, *J* = 31.05 Hz, **C**-CF_3_), 117.44, 120.91, 120.94, 127.17, 127.31, 127.68, 127.74, 127.91, 130.75 (Ar-CH), 140.47, 148.24 (Ar-C), 153.50 (thz. C2), 168.28 (CONH), 170.87 (thiaz. C4). Anal. Calcd. for C_18_H_14_F_3_N_3_O_3_S (409.383): C, 52.81; H, 3.45; N, 10.26; S, 7.83. Found: C, 52.48; H, 3.29; N, 10.38; S, 7.65.

#### (RS)-N'-[3–(3-fluorophenyl)-5-methyl-4-oxo-1,3-thiazolidin-2-ylidene]-2-hydroxy-2-phenylacetohydrazide (4a)

White powder, m.p. 173–175 °C, 80% yield; IR (KBr) (υ, cm^−1^), 3285 (OH/NH), 1696, 1764 (C=O); ^1^H-NMR (DMSO-d_6_, 500 MHz) δ (ppm): 1.54 (3H, d, *J =* 6.83 Hz, C_5_-CH_3_), 4.46 (1H, q, *J =* 6.83 Hz, H5-thz), 5.18 (1H, d, *J =* 1.46 Hz, C*H*OH), 6.47 (1H, brs, CHO*H*), 6.63 (1H, brs, Ar-H), 6.71 (1H, d, *J =* 7.81 Hz, Ar-H), 6.97 (1H, td, *J =* 8.54, 2.11 Hz, Ar-H), 7.28–7.33 (3H, m, Ar-H), 7.40 (1H, q, *J =* 7.65 Hz, Ar-H), 7.54 (2H, d, *J =* 7.32 Hz, Ar-H), 10.89 (1H, brs, CONH). Anal. Calcd. for C_18_H_16_FN_3_O_3_S (373.402): C, 57.90; H, 4.32; N, 11.25; S, 8.59. Found: C, 57.59; H, 4.30; N, 11.18; S, 8.41.

#### (RS)-N'-[3–(4-fluorophenyl)-5-methyl-4-oxo-1,3-thiazolidin-2-ylidene]-2-hydroxy-2-phenylacetohydrazide (4b)

White powder, m.p. 163–165 °C, 75% yield; IR (KBr) (υ, cm^−1^), 3263 (OH/NH), 1695, 1764 (C=O); ^1^H-NMR (DMSO-d_6_, 500 MHz) δ (ppm): 1.53 (3H, d, *J =* 7.32 Hz, C_5_-CH_3_), 4.43 (1H, q, *J =* 6.83 Hz, H5-thz), 5.16 (1H, s, C*H*OH), 6.50 (1H, s, CHO*H*), 6.87 (2H, q, *J =* 4.88 Hz, Ar-H), 7.19 (2H, td, *J =* 6.84, 1.95 Hz, Ar-H), 7.29–7.34 (3H, m, Ar-H), 7.53 (2H, d, *J =* 7.32 Hz, Ar-H), 10.90 (1H, s, CONH). Anal. Calcd. for C_18_H_16_FN_3_O_3_S (373.402): C, 57.90; H, 4.32; N, 11.25; S, 8.59. Found: C, 57.55; H, 4.05; N, 11.10; S, 8.23.

#### (RS)-N'-[3–(3-chlorophenyl)-5-methyl-4-oxo-1,3-thiazolidin-2-ylidene]-2-hydroxy-2-phenylacetohydrazide (4c)

Ivory powder, m.p. 197–198 °C, 84% yield; IR (KBr) (υ, cm^−1^), 3235 (OH/NH), 1698, 1764 (C=O); ^1^H-NMR (DMSO-d_6_, 500 MHz) δ (ppm): 1.54 (3H, d, *J =* 6.83 Hz, C_5_-CH_3_), 4.47 (1H, q, *J =* 7.32 Hz, H5-thz), 5.18 (1H, s, C*H*OH), 6.50 (1H, brs, CHO*H*), 6.83 (2H, d, *J =* 6.83 Hz, Ar-H), 7.19 (1H, d, *J =* 7.32 Hz, Ar-H), 7.29–7.33 (3H, m, Ar-H), 7.39 (1H, t, *J =* 8.30 Hz, Ar-H), 7.53 (2H, d, *J =* 6.83 Hz, Ar-H), 10.90 (1H, s, CONH). ^13^C-NMR (proton decoupled, DMSO-d_6_, 150 MHz) δ (ppm): 19.26 (thz. C5-**C**H_3_), 39.69 (thz. C5), 72.97 (CH-OH), 119.54, 120.58, 124.25, 127.22, 127.70, 127.93, 131.05, 133.49 (Ar-CH), 140.40, 148.85, 149.04 (Ar-C), 152.11 (thz. C2), 170.68 (CONH), 171.61 (thz. C4). Anal. Calcd. for C_18_H_16_ClN_3_O_3_S (389.857): C, 55.45; H, 4.14; N, 10.78; S, 8.23. Found: C, 55.18; H, 3.97; N, 10.90; S, 8.58.

#### (RS)-N'-[3–(4-chlorophenyl)-5-methyl-4-oxo-1,3-thiazolidin-2-ylidene]-2-hydroxy-2-phenylacetohydrazide (4d)

White powder, m.p. 183–185 °C, 75% yield; IR (KBr) (υ, cm^−1^), 3264 (OH/NH), 1693, 1762 (C=O); ^1^H-NMR (DMSO-d_6_, 500 MHz) δ (ppm): 1.54 (3H, d, *J =* 7.32 Hz, C_5_-CH_3_), 4.46 (1H, q, *J =* 7.32 Hz, H5-thz), 5.18 (1H, s, C*H*OH), 6.50 (1H, brs, CHO*H*), 6.87 (2H, d, *J =* 8.30 Hz, Ar-H), 7.28–7.39 (3H, m, Ar-H), 7.40–7.42 (2H, m, Ar-H), 7.52 (2H, d, *J =* 7.32 Hz, Ar-H), 10.87 (1H, s, CONH). Anal. Calcd. for C_18_H_16_ClN_3_O_3_S (389.857): C, 55.45; H, 4.14; N, 10.78; S, 8.23. Found: C, 55.21; H, 4.24; N, 10.60; S, 7.89.

#### (RS)-N'-[3–(3-bromophenyl)-5-methyl-4-oxo-1,3-thiazolidin-2-ylidene]-2-hydroxy-2-phenylacetohydrazide (4e)

Ivory powder, m.p. 199–200 °C, 87% yield; IR (KBr) (υ, cm^−1^), 3248 (OH/NH), 1694, 1765 (C=O); ^1^H-NMR (DMSO-d_6_, 500 MHz) δ (ppm): 1.54 (3H, d, *J =* 7.32 Hz, C_5_-CH_3_), 4.47 (1H, q, *J =* 7.32 Hz, H5-thz), 5.19 (1H, s, C*H*OH), 6.47 (1H, brs, CHO*H*), 6.87–6.88 (1H, m, Ar-H), 6.98 (1H, brs, Ar-H), 7.29–7.34 (5H, m, Ar-H), 7.53 (2H, d, *J =* 7.32 Hz, Ar-H), 10.87 (1H, brs, CONH). ^13^C-NMR (proton decoupled, DMSO-d_6_, 150 MHz) δ (ppm): 19.26 (thz. C_5_-**C**H_3_), 39.69 (thz. C_5_), 72.97 (CH-OH), 119.92, 121.88, 123.43, 127.12, 127.22, 127.70, 127.93, 131.33 (Ar-CH), 140.39, 148.97, 149.17 (Ar-C), 152.12 (thz. C2), 170.69 (CONH), 171.63 (thz. C4). Anal. Calcd. for C_18_H_16_BrN_3_O_3_S (434.308): C, 49.78; H, 3.71; N, 9.68; S, 7.38. Found: C, 49.86; H, 3.64; N, 9.35; S, 7.20.

#### (RS)-N'-[3–(4-bromophenyl)-5-methyl-4-oxo-1,3-thiazolidin-2-ylidene]-2-hydroxy-2-phenylacetohydrazide (4f)

Ivory powder, m.p. 172–174 °C, yield 88%; IR (KBr) (υ, cm^−1^), 3264 (OH/NH), 1693, 1760 (C=O); ^1^H-NMR (DMSO-d_6_, 500 MHz) δ (ppm): 1.53 (3H, d, *J =* 7.32 Hz, C_5_-CH_3_), 4.46 (1H, q, *J =* 6.83 Hz, H5-thz), 5.18 (1H, s, C*H*OH), 6.50 (1H, brs, CHO*H*), 6.82 (2H, d, *J =* 7.81 Hz, Ar-H), 7.31–7.34 (4H, m, Ar-H), 7.52–7.55 (3H, m, Ar-H), 10.87 (1H, s, CONH). Anal. Calcd. for C_18_H_16_BrN_3_O_3_S (434.308): C, 49.78; H, 3.71; N, 9.68; S, 7.38. Found: C, 49.60; H, 3.46; N, 9.74; S, 7.68.

#### (RS)-N'-[3–(3-methylphenyl)-5-methyl-4-oxo-1,3-thiazolidin-2-ylidene]-2-hydroxy-2-phenylacetohydrazide (4g)

White powder, m.p. 179–180 °C, 75% yield; IR (KBr) (υ, cm^−1^), 3295 (OH/NH), 1698, 1761 (C=O); ^1^H-NMR (DMSO-d_6_, 500 MHz) δ (ppm): 1.53 (3H, d, *J =* 6.83 Hz, C_5_-CH_3_), 2.29 (3H, s, -CH_3_), 4.41 (1H, q, *J =* 6.83 Hz, H5-thz), 5.17 (1H, s, C*H*OH), 6.49 (1H, brs, CHO*H*), 6.66 (2H, d, *J =* 5.86 Hz, Ar-H), 6.95 (1H, d, *J =* 7.81 Hz, Ar-H), 7.24 (1H, t, *J =* 7.81 Hz, Ar-H), 7.29–7.34 (3H, m, Ar-H), 7.54 (2H, d, *J =* 6.83 Hz, Ar-H), 10.86 (1H, brs, CONH). ^13^C-NMR (HSQC-2 D, DMSO-d_6_, 125 MHz) δ (ppm): 19.90 [thz. C_5_-**C**H_3_ – 1.53 (3H, d, *J =* 6.83 Hz, C_5_-C**H_3_**)], 40.11 [thz. C_5_ – 4.41 ppm (1H, q, *J =* 6.83 Hz, SCH)], 73.72 [**C**HOH – 5.17 ppm (1H, s, C**H**OH)], 118.31 [Ar-**C**H – 6.66 ppm (2H, d, *J* = 5.86 Hz, Ar-H)], 122.01 [Ar-**C**H – 6.66 ppm (2H, d, *J* = 5.86 Hz, Ar-H)], 125.77 [Ar-**C**H – 6.95 ppm (1H, d, *J =* 7.81 Hz, Ar-H)], 127.99 [Ar-**C**H – 7.54 (2H, d, *J =* 6.83 Hz, Ar-H)], 128.3599 [Ar-**C**H – 7.29–7.34 (3H, m, Ar-H)], 129.79 [Ar-**C**H – 7.24 (1H, t, *J =* 7.81 Hz, Ar-H)]. ^13^C-NMR (proton decoupled, DMSO-d_6_, 150 MHz) δ (ppm): 19.10 (thz. C_5_-**C**H_3_), 20.94 (Ar-**C**H_3_), 39.39 (thz. C5), 72.97 (CH-OH), 117.58, 121.27, 125.06, 127.30, 127.64, 127.72, 127.92, 129.08 (Ar-CH), 138.65, 140.51, 147.36 (Ar-C), 150.30 (thz. C2), 170.65 (CONH), 171.60 (thz. C4). LC/MS: *m/z* 368 (M-H)^−^. Anal. Calcd. for C_19_H_19_N_3_O_3_S (369.439): C, 61.77; H, 5.18; N, 11.37; S, 8.68. Found: C, 61.75; H, 4.89; N, 11.45; S, 8.79.

#### (RS)-N'-[3–(4-methylphenyl)-5-methyl-4-oxo-1,3-thiazolidin-2-ylidene]-2-hydroxy-2-phenylacetohydrazide (4h)

Ivory powder, m.p. 164–167 °C, 80% yield; IR (KBr) (υ, cm^−1^), 3265 (OH/NH), 1694, 1759 (C=O); ^1^H-NMR (DMSO-d_6_, 500 MHz) δ (ppm): 1.52 (3H, d, *J =* 6.83 Hz, C_5_-CH_3_), 4.39 (1H, q, *J =* 7.32 Hz, H5-thz), 5.15 (1H, s, C*H*OH), 6.75 (1H, s, CHO*H*), 7.15 (2H, d, *J =* 8.30 Hz, Ar-H), 7.27–7.34 (4H, m, Ar-H), 7.44 (1H, t, *J =* 7.32 Hz, Ar-H), 7.54 (2H, d, *J =* 6.83 Hz, Ar-H), 10.90 (1H, s, CONH). Anal. Calcd. for C_19_H_19_N_3_O_3_S (369.439): C, 61.77; H, 5.18; N, 11.37; S, 8.68. Found: C, 61.48; H, 5.25; N, 11.20; S, 8.45.

#### (RS)-N'-[3–(4-methoxyphenyl)-5-methyl-4-oxo-1,3-thiazolidin-2-ylidene]-2-hydroxy-2-phenylacetohydrazide (4i)

White powder, m.p. 155–157 °C, 78% yield; IR (KBr) (υ, cm^−1^), 3272 (OH/NH), 1692, 1762 (C=O); ^1^H-NMR (DMSO-d_6_, 500 MHz) δ (ppm): 1.60 (3H, d, *J =* 7.32 Hz, C_5_-CH_3_), 3.78 (3H, s, -OCH_3_), 4.47 (1H, q, *J =* 7.32 Hz, H5-thz), 5.03 (1H, s, C*H*OH), 6.81 (1H, brs, CHO*H*), 7.01 (2H, dd, *J =* 6.83, 2.44 Hz, Ar-H), 7.21 (2H, dd, *J =* 6.83, 2.44 Hz, Ar-H), 7.25–7.28 (1H, m, Ar-H), 7.32 (2H, td, *J =* 7.81, 1.47 Hz, Ar-H), 7.42 (2H, d, *J =* 7.32 Hz, Ar-H), 10.86 (1H, brs, CONH). Anal. Calcd. for C_19_H_19_N_3_O_4_S (385.438): C, 59.21; H, 4.97; N, 10.90; S, 8.32. Found: C, 59.53; H, 5.16; N, 11.03; S, 7.95.

#### (RS)-N'-[3–(4-nitrophenyl)-5-methyl-4-oxo-1,3-thiazolidin-2-ylidene]-2-hydroxy-2-phenylacetohydrazide (4j)

Yellow crystal, m.p. 166.5–168 °C, 64% yield; IR (KBr) (υ, cm^−1^), 3444, 3288 (OH/NH), 1684, 1718 (C=O); ^1^H-NMR (DMSO-d_6_, 500 MHz) δ (ppm): 1.36 (3H, d, *J =* 6.83 Hz, C_5_-CH_3_), 4.53 (1H, q, *J =* 6.83 Hz, H5-thz), 5.19 (1H, brs, C*H*OH), 6.51 (1H, brs, CHO*H*), 7.77 (4H, d, *J = 7*.81 Hz, Ar-H), 8.21 (5H, d, *J =* 7.03 Hz, Ar-H), 11.55 (1H, brs, CONH). Anal. Calcd. for C_18_H_16_N_4_O_5_S (400.410): C, 53.99; H, 4.03; N, 13.99; S, 8.01. Found: C, 54.05; H, 3.87; N, 13.80; S, 8.12.

#### (RS)-N'-[3–(4-trifluoromethylphenyl)-5-methyl-4-oxo-1,3-thiazolidin-2-ylidene]-2-hydroxy-2-phenylacetohydrazide (4k)

White powder, m.p. 166–167 °C, 86% yield; IR (KBr) (υ, cm^−1^), 3262 (OH/NH), 1697, 1766 (C=O); ^1^H-NMR (DMSO-d_6_, 500 MHz) δ (ppm): 1.55 (3H, d, *J =* 7.26 Hz, C_5_-CH_3_), 4.49 (1H, q, *J =* 7.26 Hz, H5-thz), 5.19 (1H, s, C*H*OH), 6.51 (1H, brs, CHO*H*), 7.08 (1H, brs, Ar-H), 7.18 (1H, d, *J =* 7.78 Hz, Ar-H), 7.29–7.33 (3H, m, Ar-H), 7.50 (1H, d, *J =* 7.78 Hz, Ar-H), 7.55 (2H, d, *J =* 7.26 Hz, Ar-H), 7.61 (1H, t, *J =* 7.78 Hz, Ar-H), 10.92 (1H, brs, CONH). ^13^C-NMR (HSQC-2D, DMSO-d_6_, 125 MHz) δ (ppm): 19.74 [thz. C_5_-**C**H_3_ – 1.55 (3H, d, *J =* 7.26 Hz, C_5_-C**H_3_**)], 40.48 [thz. C_5_ – 4.49 ppm (1H, q, *J =* 7.26 Hz, SCH)], 73.73 [**C**HOH – 5.19 ppm (1H, s, C**H**OH)], 118.09 [Ar-**C**H – 7.08 (1H, brs, Ar-H)], 121.64 [Ar-**C**H – 7.50 ppm (1H, d, *J =* 7.78 Hz, Ar-H)], 125.51 [Ar-**C**H – 7.18 (1H, d, *J =* 7.78 Hz, Ar-H)], 127.93 [Ar-**C**H – 7.55 (2H, d, *J =* 7.26 Hz, Ar-H)], 128.61 [Ar-**C**H – 7.29–7.33 (3H, m, Ar-H)], 131.39 [Ar-**C**H – 7.61 (1H, t, *J =* 7.78 Hz, Ar-H)]. ^13^C-NMR (proton decoupled, DMSO-d_6_, 150 MHz) δ (ppm): 19.11 (thz. C_5_-**C**H_3_), 72.98 (CH-OH), 123.89 (q, *J* = 271 Hz, CF_3_), 130.06 (q, *J* = 31.05 Hz, **C**-CF_3_), 117.30, 117.41, 120.94, 120.97, 127.22, 127.67, 127.90, 130.70 (Ar-CH), 140.47, 148.26 (Ar-C), 152.49 (thz. C2), 170.80 (CONH), 171.62 (thz. C4). Anal. Calcd. for C_19_H_16_F_3_N_3_O_3_S (423.410): C, 53.90; H, 3.81; N, 9.92; S, 7.57. Found: C, 53.55; H, 3.69; N, 9.9.54; S, 7.62.

### hCA IX enzyme inhibition assays

A stopped-flow instrument (SX.18 MV-R Applied Photophysics model) was used for assaying the CA-catalysed CO_2_ hydration activity^35^. Inhibitor and enzyme were pre-incubated for 15 min for allowing the complete formation of the enzyme-inhibitor adduct. IC_50_ values were obtained from dose response curves working at seven different concentrations of test compound (from 0.1 nM to 50 µM), by fitting the curves using PRISM (www.graphpad.com) and non-linear least squares methods, the obtained values representing the mean of at least three different determinations. The inhibition constants (*K*_I_) were derived from the IC_50_ values by using the Cheng-Prusoff equation, as follows: *K*_I_ = IC_50_/(1 + [S]/Km) where [S] represents the CO_2_ concentration at which the measurement was carried out, and Km the concentration of substrate at which the enzyme activity is at half maximal. All enzymes used were recombinant, produced in *Escherichia*  *coli* as reported earlier^36^. The concentration of hCA IX used in the assay was 12.1 nM.

### Preparation of compound series 3 and 4 for docking studies

The three-dimensional structures of all ligands, including all possible stereoisomers, were prepared (MOE software package, v2016.08, Chemical Computing Group, Inc, Montreal, Canada). Afterwards, the ligands were energy minimised using the MMFF94x force field.

### Preparation of hCA IX structure

The crystal structure of the catalytic domain of hCA IX in complex with acetazolamide (pdb: 3iai; 2.20 Å) was obtained from the RCSB protein databank. The structure was protonated using the Protonate3D tool^37^ of the MOE software package and subsequently the obtained structure was energy-minimised using the AMBER12:EHT force field. The protein atoms of subunit A and the corresponding active site zinc ion were retained and all other atoms were omitted.

### Docking protocol

The GOLD Suite software package (v5.6.1, CCDC, Cambridge, UK) and the ChemScore scoring function were used to dock the compounds into the hCA IX structures (25 dockings per ligand). The binding pocket was defined as all residues within 13 Å of a centroid corresponding to the location of the acetazolamide C2 atom. The best three docked poses were retained for each ligand.

### Molecular dynamics simulations

All molecular dynamics simulations were performed using the NAMD software package (v2.12, Theoretical and Computational Biophysics group, NIH Center for Macromolecular Modeling and Bioinformatics, The Beckman Institute, University of Illinois at Urbana-Champaign)^38^. The select docked poses (ligand-enzyme complexes) were first placed into the centre of a box with periodic boundary conditions (minimal distance of 10 Å between protein and boundary). Afterwards, both water molecules (Tip3) and counter ions (NaCl) were added to generate a solvated and neutral system. After a steepest-descent energy minimisation (AMBER12:EHT), the system was first heated from 0 to 300 K during 100 ps followed by an 100 ps equilibration simulation (position restraints on all protein and ligand heavy atoms). Finally, the system was simulated for 1 ns at constant temperature (300 K, Langevin, default values) and pressure (1 bar, Nosé-Hoover Langevin, default values), without any position restrains. The only restraints applied were distance restraints to keep the zinc ion in the correct orientation towards His94, His96, and His119 (distance restraints between Zn and N atom of histidine: 1.8 Å; default settings). The timestep was set to 0.002 fs and all bonds were constrained using the ShakeH algorithm.

## Results and discussion

### Chemistry

**Scheme 1.**
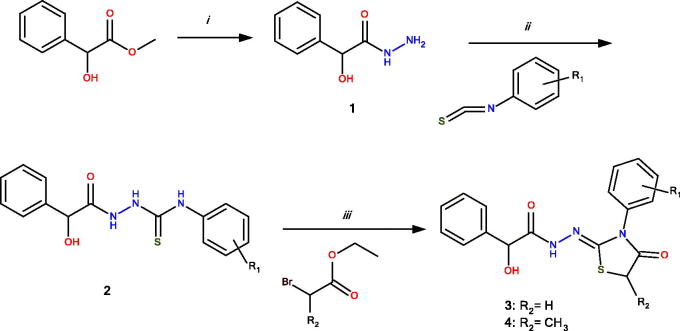
General synthesis of **3**a-o and **4**a-k. Reagents and conditions: (i) hydrazine hydrate, EtOH, reflux, 6 h; (ii) EtOH, reflux, 3 h; and (iii) sodium acetate, anhydrous EtOH, reflux, 3 h.

The chemical synthesis of **3a-o** and **4a-k** compounds is outlined in Scheme 1. The synthesis of several intermediate thiosemicarbazide derivatives except **2g** and **2h** were previously reported elsewhere^32^^,^^39^. 4-Thiazolidinones were prepared starting from 2-hydroxy-2-phenylacetohydrazide (**1**) which afforded intermediate thiosemicarbazides (**2**) on reaction with aryl isothiocyanates. The thiosemicarbazides in turn furnished **3** and **4** with ethyl α-bromoacetate and ethyl α-bromopropionate, respectively.

The structures of **2g**, **2h**, **3a-o**, and **4a-k** were confirmed by analytical and spectral (IR, ^1^H NMR, ^13^C-NMR (proton decoupled), HSQC-2D, and LCMS-APCI) data.

The IR spectra exhibited O-H/N-H and C=O bands in the 3226–3567 cm^−1^ and 1684–1705 cm^−1^ regions attributed to the common CONH functions of **2**, **3**, and **4**^34^^,^^40^. Observation of new endocyclic C=O bands (1718–1766 cm^−1^) characteristic for such structures besides C=O amide bands (1684–1705 cm^−1^) in the IR spectra of **3** and **4** supported the aimed cyclisation^34^^,^^40^.

The ^1^H-NMR spectra of **3** and **4** displayed two singlets and two quartettes attributed to the methylene (SCH_2_) and methane (SC*H*CH_3_) ring protons at 5-position of the 4-thiazolidinone system at about δ 4.13–4.24 and 4.39–4.53 ppm, respectively. The C-OH and CONH protons were observed at about δ 6.46–6.81 and δ 10.81–11.55 ppm, respectively^34^^,^^40^.

HSQC 2D NMR experiments of compounds **3f**, **3m**, **4g**, and **4k** allowed explicit assignments for the proton and carbon chemical shifts. The spectra substantiated the expected conversion and revealed the typical 4-thiazolidinone C5 (compound **3**), and C5 (compound **4**) resonances at δ 30.48–30.63, and 40.11–40.48 ppm, respectively^40^. Existence of cross peaks connecting C5 (δ 30.48–30.63 ppm) with the singlet at δ 4.13–4.18 ppm provided evidence for unambiguous assignment for compounds **3f** and **3m**. Existence of cross peaks connecting C5-CH_3_ (δ 19.74–21.65 ppm) with the doublet at δ 1.53–1.55 ppm and C5 (δ 40.11–40.48 ppm) with the quartette at δ 4.41–4.49 ppm allowed for unambiguous assignment for compounds **4g** and **4k**. ^13^C NMR data of compounds **3d**, **3f**, **3m**, **3o**, **4c**, **4e**, **4g**, and **4k** also support the structure of 4-thiazolidinone ring via C2, C4, and C5 resonances that appeared at 150.30–153.75, 170.50–171.63, and 29.76–30.05 ppm (19.10–19.26 ppm for compound **4**), respectively^41^. The CONH resonance of compounds **3** and **4** was observed at δ 168.22–170.80 ppm^40^^,^^41^.

In the mass spectra of **3c** and **4g** (M-H)^−^ peaks were observed which confirmed their molecular weights. Further spectral details have already been presented in the “Materials and methods” section.

### hCA IX enzyme inhibition studies

A small collection of 26 thiazolidinone compounds have been tested in enzyme inhibition assays against hCA IX and 17 compounds were identified with an inhibition constant (*K*_I_) lower than 25 μM (Table 1). A subset of five compounds show *K*_I_ values lower than 10 μM (**3d**: 1.4 μM; **3f**: 1.2 μM; **3o**: 1.1 μM; **4c**: 2.4 μM; **4e**: 7.7 μM). The measured *K*_I_ values for these ligands are at least 44-fold lower compared to the *K*_I_ value of the potent hCA inhibitor acetazolamide (25 nM). However, it should be noted that these compounds do not contain a sulphonamide moiety that functions as a ZBG, which is present in many inhibitors of hCAs.

**Table 1.** Inhibition constants (*K*_I;_ μM) for hCA IX of compound series **3** and **4**.

**3,4**			
cmp	R_1_	R_2_	hCA IX
**3a**	2-F	H	22.8
**3b**	3-F	H	>100
**3c**	4-F	H	18.2
**3d**	2-Cl	H	1.4
**3e**	3-Cl	H	13.2
**3f**	4-Cl	H	1.2
**3g**	2-Br	H	>100
**3h**	3-Br	H	17.3
**3i**	4-Br	H	>100
**3j**	2-CH_3_	H	>100
**3k**	3-CH_3_	H	13.7
**3l**	4-CH_3_	H	>100
**3m**	4-OCH_3_	H	20.4
**3n**	4-NO_2_	H	22.5
**3o**	3-CF_3_	H	1.1
**4a**	3-F	CH_3_	16.7
**4b**	4-F	CH_3_	11.4
**4c**	3-Cl	CH_3_	2.4
**4d**	4-Cl	CH_3_	>100
**4e**	3-Br	CH_3_	7.7
**4f**	4-Br	CH_3_	12.5
**4g**	3-CH_3_	CH_3_	>100
**4h**	4-CH_3_	CH_3_	>100
**4i**	4-OCH_3_	CH_3_	>100
**4j**	4-NO_2_	CH_3_	21.3
**4k**	3-CF_3_	CH_3_	17.3
**AAZ**	–	–	0.025

Mean from three different assays, by a stopped flow technique (errors were in the range of ±5–10% of the reported values).

It is difficult to draw conclusive structure-activity relationships from these data since the *K*_I_ values are relatively close to each other.

Compounds **3n** and **4j** show *K*_I_ values of 22.5 and 21.3 μM, respectively. These two compounds have nitro groups on the para positions of their phenyl moieties. This may indicate the presence of a direct interaction between the nitro oxygen atoms and the zinc ion of the hCA IX active site. The other compounds may form interactions with the zinc ion via their hydroxyl or carbonyl groups.

Docking studies and molecular dynamics simulations have been performed to suggest possible binding poses for these compounds.

### Docking studies into the active site of hCA IX

All 26 thiazolidinone-containing molecules were docked into the active site of hCA IX, with and without a zinc-bound water molecule.

**Figure 1.**
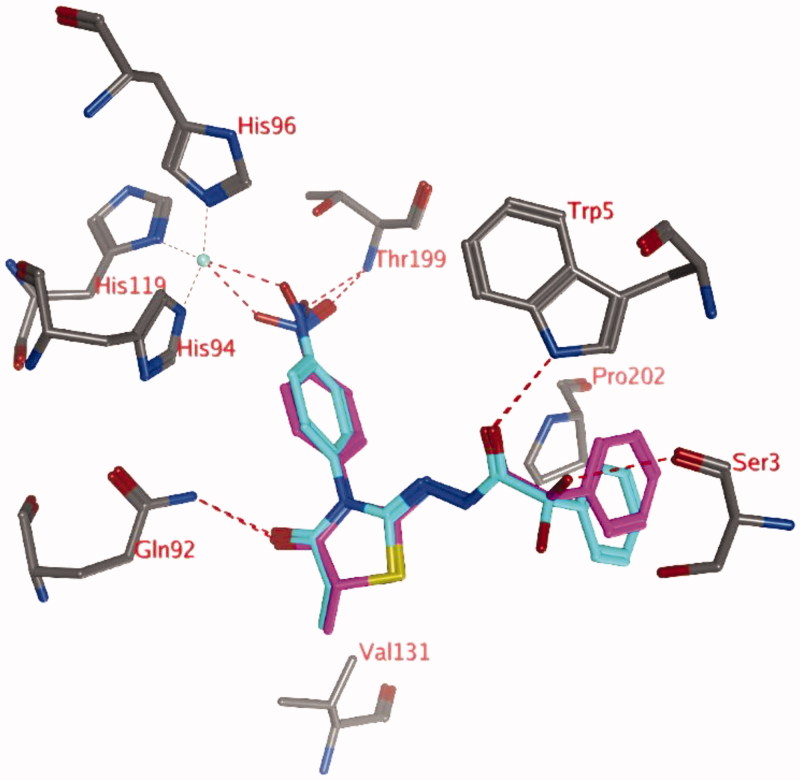
Representation of docking pose 1, showing compound **4j** (purple; OH in R isomer; turquoise OH in S isomer) in the active site of hCA IX. Hydrogen bonds and interactions to the Zn^2+^-ion are depicted in red dashed lines. A similar docked pose has been obtained for compound **3n**.

Docking studies without a zinc-bound water molecule suggests that only compounds with para-nitro substituents (compounds **3n** and **4j**; pose 1) may interact directly with the active site zinc ion (Figure 1). The carbonyl group of the thiazolidinone ring forms a hydrogen bond with the side chain of Gln92, while the other carbonyl group forms a hydrogen bond to the side chain of Trp5. The unsubstituted phenyl group forms hydrophobic interactions with the side chain of Pro202. An additional hydrogen bond is formed between the hydroxyl group (R isomer) with the backbone of Ser3. In the S isomer, this hydroxyl group may interact with the backbone carbonyl group of Pro201 or point to the solvent. The methyl group at the R2 position (R isomer) may form a hydrophobic interaction with the side chain of Val131.

**Figure 2.**
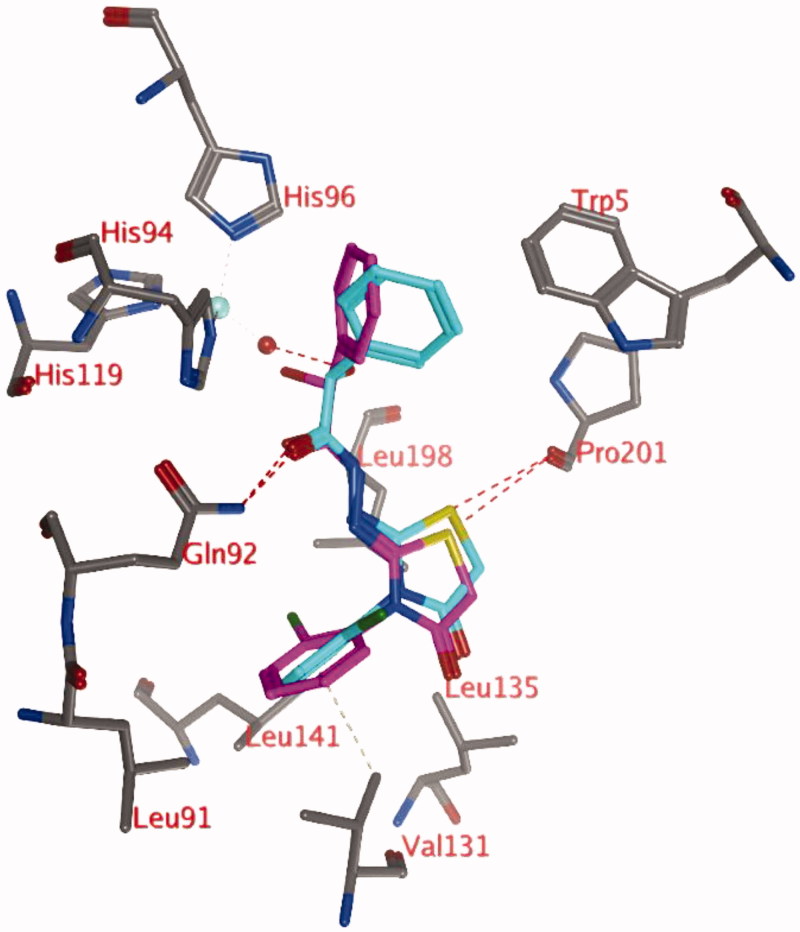
Representation of docking pose 2, showing compound **3d** (turquoise; OH in R isomer; purple OH in S isomer) in the active site of hCA IX. Hydrogen bonds and interactions to the Zn^2+^-ion are depicted in red dashed lines.

The docking studies with a zinc-bound water molecule in the active site suggest docked poses in which the ligand interacts directly with the water molecule (pose 2; Figure 2).

In pose 2, the unsubstituted phenyl moiety forms a hydrophobic interaction with the side chain of His94 (Figure 2). The carbonyl group forms a hydrogen bond to the side chain of Gln92 and the substituted phenyl ring is located in a hydrophobic pocket formed by Leu91, Val121, Val131, Leu135, Leu141, and Leu198. The ligands sulphur atom may form interactions with the backbone carbonyl of Pro201. The thiazolidinone carbonyl group is solvent exposed. The hydroxyl group of the ligand in the R isomer (turquoise, Figure 2) forms a direct hydrogen bond with the zinc-bound water molecule, while this is not observed for the hydroxyl group in the S isomer (purple, Figure 2). However, the distance and orientation of this hydroxyl group are such that it might be possible after slight conformational changes in this area.

Docking pose 2, with the hydroxyl group in either S or R orientation, was observed for all compounds **3** and **4**, as the hydrophobic cavity formed by Leu91, Val121, Val131, Leu135, Leu141, and Leu198 seems to be large enough to accept the substituted phenyl group of the ligand.

### Molecular dynamics simulations

Molecular dynamics simulations have been performed to investigate whether the suggested docked poses 1 and 2 would be stable during molecular dynamics simulations. To this end, the ligand-enzyme complex was first placed into a box with periodic boundary conditions. Afterwards, both water molecules and counter ions (NaCl) were added to generate a solvated and neutral system. After a steepest-descent energy minimisation (AMBER12:EHT), the system was first heated from 0 to 300 K during 100 ps followed by a 100-ps equilibration simulation (position restraints on all protein and ligand heavy atoms). Finally, the system was simulated for 1 ns at constant temperature (300 K) and pressure (1 bar), without any position restrains. The only restraints applied were dihedral and distance restraints to keep the zinc ion in the correct orientation towards His94, His96, and His119.

#### MD simulation of hCA IX-4j complex (pose 1)

**Figure 3.**
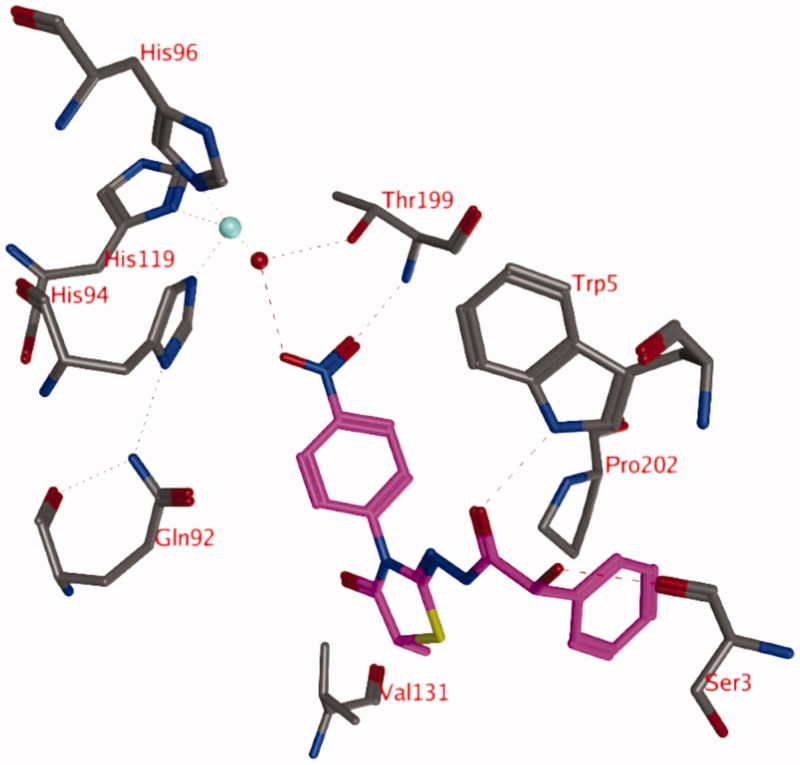
Snapshot of docking pose 1 after a 1 ns MD simulation, showing compound **4j** (OH in R isomer) in the active site of hCA IX. Hydrogen bonds and interactions to the Zn^2+^-ion are depicted in red dashed lines.

The simulation of compound **4j** (with hydroxyl in R isomer) in the active site of hCA IX (docked pose 1; purple; Figure 1) indicates that the interaction between the oxygen atom of the nitro group and the zinc ion is not stable. The direct interaction is lost and a water molecule comes in between (Figure 3). The newly obtained pose of compound **4j** is remarkably similar to its original docked pose (0 ps), with the exception of the bridging water molecule and the absence of an interaction with Gln92 (Figure 3). Hydrogen bonds are being formed with the side chain of Trp5 (84.3% of 1 ns simulation) and Thr199 (81.8% of 1 ns simulation) and the backbone of Ser3 (65.4% of 1 ns simulation).

A similar result has been observed for the molecular dynamics simulation of compound **4j** with the hydroxyl group in the S isomer (turquoise; Figure 1). Again the nitro group forms interactions with a zinc-bound water molecule and the backbone of Thr199 (89.2% of 1 ns simulation) or Thr200 (9.2% of 1 ns simulation). In addition, hydrogen bonds are formed with the backbone of Ser3 (12.2% of 1 ns simulation) and the side chain of Trp5 (86.1% of 1 ns simulation).

#### MD simulation of hCA IX-3d complex (pose 2)

**Figure 4.**
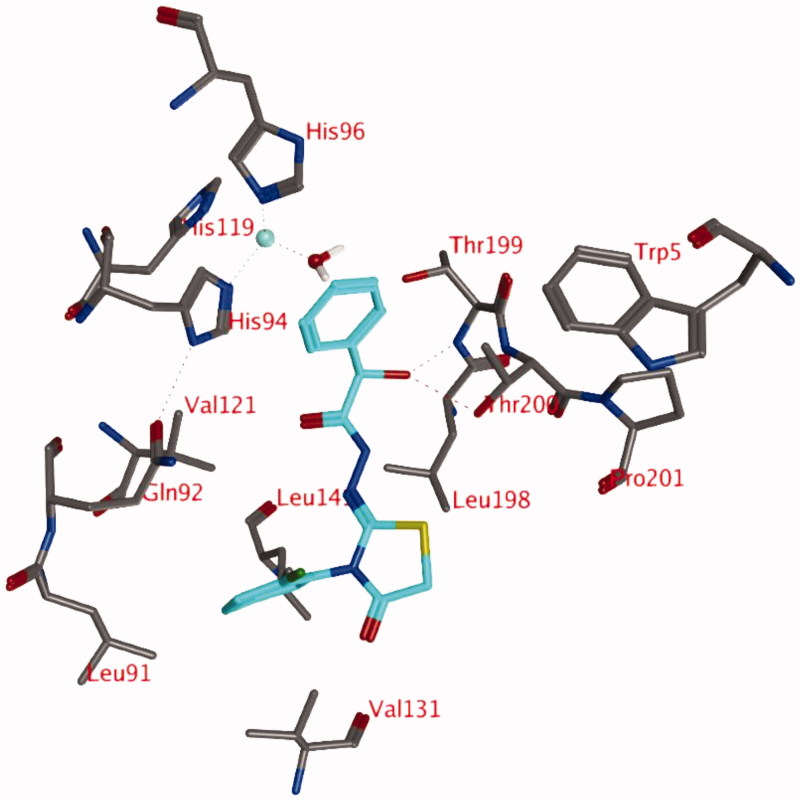
Snapshot of docking pose 1 after a 1 ns MD simulation, showing compound **3d** (OH in R isomer) in the active site of hCA IX. Hydrogen bonds and interactions to the Zn^2+^-ion are depicted in red dashed lines.

The simulation of compound **3d** (R) reveals that the hydrogen bond between the hydroxyl group of the ligand (R isomer) and the zinc-bound water molecule is not stable and this bond is lost very early in the simulation (Figure 4). New hydrogen bonds are formed between the ligand and the backbone of Thr199 (69.3% of 1 ns simulation) or the side chain of Thr200 (45.0% of 1 ns simulation). An additional hydrogen bond is formed between the ligand and the side chain of Gln92 (16.0% of 1 ns simulation). One of the hydrogen atoms of the zinc-bound water molecule projects to the centroid of the unsubstituted phenyl moiety of the ligand. In addition, this unsubstituted phenyl group forms hydrophobic interactions with His94. The substituted phenyl group is located in the hydrophobic pocket formed by the side chains of Leu91, Val121, Val131, and Leu141. The two carbonyl groups and the sulphur atom form hydrogen bonds with other water molecules that have entered the active site.

The start conformation for the simulation of compound **3d** (hydroxyl in S isomer; Figure 2, purple) indicates that no hydrogen bond between the ligand and the zinc-bound water molecule is formed. The hydrogen bond with the side chain of Gln92 seems to be stable (97.7% of 1 ns simulation), whereas the interaction between the ligand sulphur and the backbone carbonyl of Pro201 is less frequently observed (4.6% of 1 ns simulation). Occasionally, the ligand forms a hydrogen bond with the side chain of Thr200 (15.7% of 1 ns simulation). The substituted phenyl group of the ligand is located in the hydrophobic pocket lined by Leu91, Val121, Val131, and Leu141 during the whole simulation.

## Conclusions

Thiazolidinone-containing compounds, without the well-known sulphonamide ZBG, inhibit the tumour-associated hCA IX enzyme with *K*_I_ values in the lower micromolar range. Docking studies in combination with molecular dynamics simulations suggested several binding poses for these compounds, in which the ligand form no direct interactions with the active site zinc ion. These structurally novel compounds, without the well-known sulphonamide ZBG, may be interesting candidates as novel classes as CAIs.
